# Molecular characterization and functional implications on mouse peripheral blood mononuclear cells of annexin proteins from *Echinococcus granulosus* sensu lato

**DOI:** 10.1186/s13071-023-05967-y

**Published:** 2023-10-06

**Authors:** Xue He, Guoqing Shao, Xiaodi Du, Ruiqi Hua, Hongyu Song, Yanxin Chen, Xiaowei Zhu, Guangyou Yang

**Affiliations:** Department of Parasitology, College of Veterinary Medicine, Sichuan Agricultural University, Chengdu, 611130 Sichuan Province People’s Republic of China

**Keywords:** *Echinococcus granulosus*, Echinococcosis, Annexins, Leukocytes, Mononuclear, Immunity

## Abstract

**Background:**

Cystic echinococcosis (CE) is a life-threatening zoonotic disease caused by the larval stage of *Echinococcus granulosus* sensu lato, which employs various strategies to evade the host immune system for survival. Recent advances have revealed the role of annexins as excretory/secretory products, providing new insights into the immune regulation by these proteins in the pathogenesis of CE.

**Methods:**

*Echinococcus granulosus* annexin B proteins *Eg*ANXB2, *Eg*ANXB18, *Eg*ANXB20, and *Eg*ANXB23 were cloned, expressed, and analyzed using bioinformatic tools. Membrane binding analysis was used to assess their bioactivity, while their immunoreactivity and tissue distribution characteristics were determined experimentally using western blotting and immunofluorescence staining, respectively. Furthermore, quantitative real-time reverse transcription PCR (qRT-PCR) was used to analyze the mRNA expression profiles of *Eg*ANXBs in different developmental stages of *E*. *granulosus*. Finally, immunofluorescence staining, cell counting kit 8 assays, flow cytometry, transwell migration assays, and qRT-PCR were used to evaluate the functional effects of r*Eg*ANXB18 and r*Eg*ANXB20 on mouse peripheral blood mononuclear cells (PBMCs).

**Results:**

In this study, we identified four *Eg*ANXBs with conserved protein structures and calcium-dependent phospholipid binding activities. r*Eg*ANXBs were recognized by serum from sheep infected with *E*. *granulosus* and distributed in the germinal layer of fertile cysts. Interestingly, transcription levels of the four *Eg*ANXBs were significantly higher in protoscoleces than in 28-day strobilated worms. Moreover, we demonstrated that r*Eg*ANXB18 and r*Eg*ANXB20 were secretory proteins that could bind to PBMCs and regulate their function. Specifically, r*Eg*ANXB18 inhibited cell proliferation and migration while promoting cell apoptosis, NO production, and cytokine profile shifting. In contrast, r*Eg*ANXB20 showed limited effects on apoptosis but inhibited NO production.

**Conclusions:**

Our findings suggested that among the four identified *Eg*ANXBs, *Eg*ANXB2 and *Eg*ANXB23 might play a pivotal role for the development of protoscoleces, while *Eg*ANXB18 and *Eg*ANXB20, as secretory proteins, appeared to participate in the host-parasite interaction by regulating the function of immune cells.

**Graphical Abstract:**

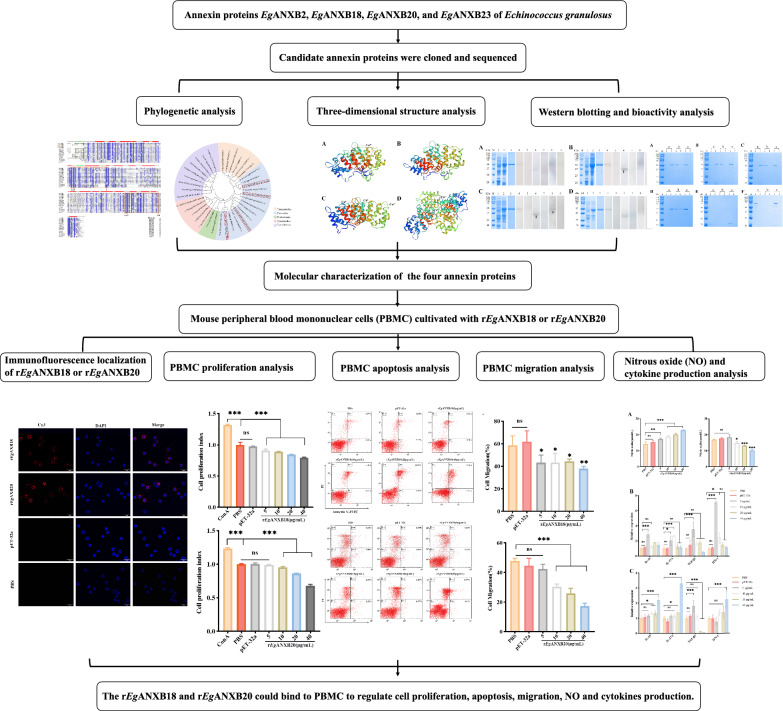

**Supplementary Information:**

The online version contains supplementary material available at 10.1186/s13071-023-05967-y.

## Background

Cystic echinococcosis (CE) is a global zoonosis caused by the larval stage of *Echinococcus granulosus* sensu lato [1]. Currently, CE still poses a significant public health challenge and causes large economic losses. This disease has been officially recognized as a neglected tropical disease by the World Health Organization because of its persistent impact on global health, affecting over 1 million people worldwide [2]. Intermediate hosts (mostly herbivores and the human as an accidental intermediate host) and definite hosts (canids) are required in the life cycle of *E. granulosus* [3]. The eggs are released from the definite hosts and subsequently ingested by the intermediate hosts, leading to the development of hydatid cysts within their internal organs, predominantly in the liver and lungs (accounting for > 90% of cases) [4]. The protoscoleces (PSCs) within cysts are known to produce a considerable quantity of excretory/secretory products (ESPs), which might play a vital role in immune evasion by the parasites [5].

Some proteins within the ESPs have been identified, and their functions have been further investigated, particularly in relation to their interactions with the host immune cells. For instance, AgB has been found to reduce the H_2_O_2_ production of human neutrophils [6] and promote the unconventional maturation of dendritic cells, resulting in reduced secretion of pro-inflammatory cytokines and the polarization of T lymphocytes towards the Th2 lineage [7]. Similarly, thioredoxin peroxidase has been found to promote the differentiation of peritoneal macrophages into alternatively activated macrophages in vitro, thereby favoring immune evasion of parasites [8]. In summary, certain proteins within the ESPs are of great significance for host-parasite interactions and highlight the potential role of these proteins in facilitating parasite growth within their hosts.

Annexins constitute an evolutionarily conserved protein superfamily with calcium-dependent phospholipid-binding properties [9]. Widely distributed from protists to eukaryotes, annexins have been confirmed to be involved in various physiological processes, such as membrane repair [10], membrane traffic [11], cell proliferation [12], apoptosis [13], and inflammation [14]. Previous studies have reported the presence of several annexins in extracellular vesicles isolated from culture medium of PSCs (*Eg*ANXB2, *Eg*ANXB18, *Eg*ANXB20, and *Eg*ANXB23), hydatid fluid (*Eg*ANXB3, *Eg*ANXB15, *Eg*ANXB33, and *Eg*ANXB38) [15–17], as well as exosomes isolated from the cyst fluid of sheep (*Eg*ANXB20) or the pulmonary hydatid fluid of patients (*Eg*ANXB2 and *Eg*ANXB20) [18, 19]. However, despite these findings, studies regarding *E. granulosus* annexins are still limited [20, 21], and their involvement in parasite immune evasion remains unknown.

In this study, we cloned and expressed *Eg*ANXB2, *Eg*ANXB18, *Eg*ANXB20, and *Eg*ANXB23 of *E. granulosus*. Then, the calcium-dependent phospholipid binding activities, secretory properties, distribution, and transcription levels of the four *Eg*ANXBs were explored. Furthermore, we investigated the regulatory effects of r*Eg*ANXB18 and r*Eg*ANXB20 on mouse peripheral blood mononuclear cells (PBMCs) to gain a better understanding of the host-parasite interaction.

## Materials and methods

### Parasites and animals

PSCs were collected from hydatid cysts isolated from the liver of sheep infected with *E. granulosus* s.l. from a slaughterhouse in Sichuan province, China. The PSCs and cyst walls were separated and treated as described in previous reports [22]. The viability and molecular genotyping of PSCs were conducted as described in our previously published work [23]. PSCs with a viability > 95% were considered for this study. Twenty-eight-day strobilated worms were obtained from the Department of Parasitology of Sichuan Agricultural University. In addition, PSCs, the germinal layer, and the strobilated worms were placed in 4% paraformaldehyde before immunolocalization. Furthermore, specific pathogen-free 6-week-old female SD rats and BALB/c mice were purchased from Chengdu Dashuo Co., Ltd (Chengdu, China).

### Cloning, expression, and purification of four *Eg*ANXBs

An RNAprep pure Tissue Kit (Tiangen, Beijing, China) was used to extract total RNA from PSCs, and cDNA was subsequently synthesized using a RevertAid First Strand cDNA Synthesis Kit (Thermo Scientific, Waltham, MA, USA). Specific primers (Additional file 1: Table S1) were designed based on the full-length coding sequences of the four *Eg*ANXBs. After PCR, the products were extracted from the gel and cloned into vector pMD19-T (Takara, Dalian, China) for sequencing. Then, the pMD19-T-*Eg*ANXBs plasmids were subcloned into vector pET-32a ( +) (Invitrogen, Waltham, MA, USA) and transformed into *Escherichia coli* BL21 or Rosetta (DE3) cells (Tiangen), followed by induction with 0.24 mg/ml isopropyl β-d-1-thiogalactopyranoside (IPTG) at 16 ℃ for 12 h. The recombinant (r)*Eg*ANXBs were purified using a Ni^2+^ affinity chromatography column (Bio-Rad, Hercules, CA, USA). The imidazole was removed, and the concentration of each protein was determined using a bicinchoninic acid (BCA) protein quantification kit (Bestbio). The purified proteins (0.5 mg/well) were assessed using 12% sodium dodecyl sulfate–polyacrylamide gel electrophoresis (SDS-PAGE), and the gels were stained with coomassie blue.

### Bioinformatic analysis

The full-length coding sequences of the four *Eg*ANXBs were obtained from the genome data of *E. granulosus* in the WormBase ParaSite database (https://parasite.wormbase.org/index.html). A comparison was made between these sequences and those we obtained here, followed by bioinformatic analysis. Expasy (http://web.expasy.org/protparam/) was used to predict the basic physicochemical properties. Protein signal peptides, transmembrane regions, and subcellular localizations were predicted using SignalP-5.0 Server (http://www.cbs.dtu.dk/Services/SignalP/), TMHMM Server (http://www.cbs.dtu.dk/services/TMHMM-2.0) and WOLFPSORT (https://wolfpsort.hgc.jp/), respectively. The secondary and three-dimensional structures were predicted using SOPMA (http://npsa-pbil.ibcp.fr/cgi-bin/npsa_automat.pl?page=npsa_sopma.html) and SWISS-MODEL (http://swissmodel.expasy.org/). The multiple sequence alignment of the four *Eg*ANXBs was analyzed with Jalview (version 2.11.1) [24], and the phylogenetic tree was constructed using the maximum likelihood method (1000 bootstrap replications) by MEGA (version 7.0.26) [25]. The sequences for analysis were from the genome data of *Echinococcus granulosus* [26].

### Ca^2+^-dependent phospholipid binding bioactivity assay

The preparation of liposomes and assessment of Ca^2+^-dependent phospholipid-binding bioactivity of the four r*Eg*ANXBs were conducted according to previously published protocols with slight modifications [20]. Briefly, three groups (a, b, and c) were included for r*Eg*ANXBs, pET32a vector protein (negative control), and bovine serum albumin (BSA) (V) (unrelated control). Both group a and b contained 30 μl of protein (0.5 mg/ml), 20 μl of liposomes (1 mg/ml) (Solarbio, Beijing, China), 30 μl of CaCl_2_ (50 mM), and 20 μl of Tris–HCl (50 mM) in a total volume of 100 μl, while group c excluded CaCl_2_. After fully mixing, all groups were incubated at 37 °C for 1 h and then centrifuged at 8000 × *g* for 10 min to separate the supernatant and precipitate. Then, 30 μl of EDTA (50 mM) and 70 μl of Tris–HCl were added to group b precipitate, incubated, centrifuged, and separated as mentioned above. All the supernatants and precipitates were subjected to 12% SDS-PAGE and Coomassie blue staining for analysis.

### Sera and polyclonal antibody preparation

Sheep sera, either positive or negative, were obtained from autopsy-confirmed infected or healthy sheep, respectively. Polyclonal antibodies against the r*Eg*ANXBs were produced by immunizing eight rats, with two rats for each protein, as previously described [27]. The pre-immune rat serum was collected as the negative serum, followed by subcutaneous injection of 0.3 mg protein fully emulsified with an equal volume of complete Freund's Adjuvant (Sigma, St. Louis, MO, USA) for the first immunization. Three booster immunizations with r*Eg*ANXBs in incomplete Freund's adjuvant were performed in the following 3 weeks, with 1-week intervals. One week after the last immunization, anti-sera were collected and purified using a Protein G Resin FF Prepacked Column (GenScript, Piscataway, NJ, USA) to obtain anti-r*Eg*ANX rat IgGs.

### Western blotting

The total proteins of PSCs were extracted using a BBprExtra total protein extraction kit (Bestbio, Shanghai, China). The extracted proteins, along with r*Eg*ANXBs and cyst fluid, were separated using 12% SDS-PAGE and transferred to Nitrocellulose Membranes (Biosharp, Hefei, China). Then, the membrane was blocked using 5% skim milk for 2 h. Sheep negative or positive serum diluted 1:100 in phosphate-buffered saline (PBS), rat negative serum, or anti-r*Eg*ANXBs rat IgG (dilution 1:400 in PBS) was added and incubated overnight at 4 °C. After washing, horseradish peroxidase (HRP)-conjugated rabbit anti-sheep IgG (dilution 1:1000 in PBS) or HRP-conjugated goat anti-rat IgG (H + L) (ABclonal, Wuhan, China) (dilution 1:2000 in PBS) was added for 2 h incubation, and the immunoreactive signals were detected using a Metal Enhanced DAB Substrate Kit (Solarbio, Beijing, China).

### Immunolocalization analysis of *Eg*ANXBs by immunofluorescence assays

Samples including PSCs, fertile cysts, non-fertile cysts, and 28-day strobilated worms were fixed in 4% paraformaldehyde for at least 24 h. Paraffin embedding and cutting into around 5-μm sections were carried out after dehydration and transparency procedures. Dewaxing, rehydration, and antigen repair (heating in 0.01 M sodium citrate solution at 95 °C for 15 min) were subsequently conducted, followed by quenching the tissue autofluorescence using an autofluorescence quenching kit (Servicebio, Wuhan, China). The sections were blocked with 5% goat serum (Solarbio) at 37 ℃ for 1 h. Rat negative serum or anti-r*Eg*ANXBs rat IgG (dilution 1:400 in PBS) was added and incubated overnight at 4 °C. Fluorescein isothiocyanate (FITC)-conjugated goat anti-rat IgG (H + L) (dilution1:2000 in PBS) (Invitrogen) was added and incubated for 1 h, followed by staining with 4′,6-diamidino-2-phenylindole for 10 min at 37 ℃ (DAPI) (Solarbio, Beijing, China). Images were acquired under a fluorescence microscope (BX61VS, Olympus, Tokyo, Japan).

### Transcription analysis of four *Eg*ANXBs

To assess the mRNA expression levels of the four *Eg*ANXBs in PSCs and 28-day strobilated worms, quantitative real-time reverse transcription PCR (qRT-PCR) was performed. Total RNA was extracted using a cell total RNA isolation kit (Foregene, Chengdu, China), and cDNA was synthesized. The specific primers are shown in Additional file 1: Table S2, and qPCR was performed using the LightCycler System (Roche, Basel, Switzerland) with TB Green Premix Ex Taq II (Takara, Shiga, Japan). The PCR procedure comprised: 30 s at 95 ℃; 40 cycles of 5 s at 95 ℃ and 30 s at 61 ℃; melting at 95 ℃ for 10 s, 65 ℃ for 5 s, 95 ℃ for 1 s. The *GAPDH* gene from *E. granulosus* (encoding glyceraldehyde-3-phosphate dehydrogenase; GenBank: KF894802) was selected as the housekeeping gene. The results were analyzed using the 2^−ΔΔCt^ method and shown as the relative expression to the gene of strobilated worm [28].

### Isolation of mouse PBMCs and endotoxin removal of r*Eg*ANXB18 and r*Eg*ANXB20 protein preparations

Blood (0.7-1 ml/per mouse) of 60 female BALB/c mice was collected from the tail vein, and the mouse PBMCs were isolated following the manufacturer's instructions of the peripheral blood mononuclear cell separation kit (TBD Science, Inc., Tianjin, China). Cell viability was detected using trypan blue staining. The cells were then resuspended in Roswell Park Memorial Institute (RPMI)1640 medium (Gibco, Grand Island, NY, USA) supplemented with 10% heat-inactivated fetal bovine serum (FBS; Newzerum, Christchurch, New Zealand) and 1% penicillin-streptomycin (HyClone, Logan, UT, USA) before being cultured at 37 ℃ in a 5% CO_2_ incubator. To avoid the effects of endotoxin on cells, endotoxins in the r*Eg*ANXB18 and r*Eg*ANXB20 preparations were removed using Endotoxin Removal Beads (Smart-Lifesciences, Changzhou, China) and tested according to the instructions of the ToxinSensor™ Chromogenic LAL Endotoxin Assay Kit (GenScript). Moreover, r*Eg*ANXBs were filtered by 0.45-μm sterile filter membranes and quantified using BCA protein quantification kit (Bestbio).

### Analysis of the binding for r*Eg*ANXB18 and r*Eg*ANXB20 to PBMCs by immunofluorescence assays

To analyze the binding of r*Eg*ANXB18 and r*Eg*ANXB20 to PBMCs, immunofluorescence staining was conducted. First, cocultivation of PBMCs (1 × 10^6^ cells/ml/well) with r*Eg*ANXB18 (10 μg), r*Eg*ANXB20 (10 μg), pET-32a vector protein (10 μg), or 10 μl PBS was conducted in 24-well plates. After 2 h incubation at 37 °C, 5% CO_2_, the cells were washed and then fixed using 4% paraformaldehyde, permeabilized with 0.25% TritonX-100 and blocked by 5% BSA for 2 h. Then, anti-r*Eg*ANXB18/r*Eg*ANXB20 rat IgG (1:200 in PBS) or rat negative serum was added and incubated at 4 °C overnight, followed by incubation with cyanine 3 (Cy3)-labeled goat anti-rat IgG (1:500 in PBS) (Beyotime, Jiangsu, China) at 37 ℃ for 2 h and stained with DAPI. The samples were observed under a fluorescence microscope.

### Proliferation, apoptosis, and migration analysis of PBMCs cultivated with rEgANXB18 or r EgANXB20

PBMCs (1 × 10^6^ cells/100 μl/well) were seeded into 96-well plates and incubated with r*Eg*ANXB18, r*Eg*ANXB20 (0.5, 1, 2, and 4 μg), pET-32a vector protein (1 μg), concanavalin A (ConA) (1 μg), or 1 μl of PBS, respectively. After incubation at 37 °C for 24 h, the proliferation of PBMCs was evaluated using the Cell Counting Kit 8 (CCK-8) reagent (Beyotime) according to the manufacturer's instructions. Cell proliferation index was calculated by the formula: OD450 of the treatment/OD450 of the control.

For apoptosis analysis, we cultivated PBMCs (1 × 10^6^ cells/ml/well) with r*Eg*ANXB18, r*Eg*ANXB20 (5, 10, 20, and 40 μg), pET-32a vector protein (10 μg), or 10 μl PBS, respectively. After incubation at 37 °C for 24 h, the cells were collected, washed, and subjected to AnnexinV-FITC (Invitrogen, Melbourne, Australia) and propidium iodide (PI) (BD Biosciences, San Jose, CA, USA) staining to detect cell apoptosis by flow cytometry (CytoFLEX, Beckman, Brea, CA, USA) [29]. Cell apoptosis rate (%) was calculated by the formula: total apoptosis rate (%) = early apoptosis rate (%) + late apoptosis rate (%).

To assess the effect of r*Eg*ANXB18/r*Eg*ANXB20 on PBMC migration, a Transwell migration assay was conducted. Specifically, cell chambers with an 8.0-µm pore size (Thermo Fisher) were gently placed in a 24-well plate; RPMI1640 medium (1300 μl) containing 10% inactivated FBS was added to the lower chamber, while 200 μl PBMCs (2 × 10^6^ cells) resuspended in RPMI1640 medium without FBS was added to the upper chamber along with r*Eg*ANXB18, r*Eg*ANXB20 (5, 10, 20, and 40 μg/ml), pET-32a vector protein (10 μg/ml), or 10 μl PBS. PBMCs were incubated for 4 h at 37 °C, and cells that migrated to the lower chamber were collected and counted using a cell counting chamber (Corning Inc., Corning, NY, USA). Cell migration rate (%) was calculated by the formula: migration rate (%) = cells that migrated to the lower chamber/ 2 × 10^6^ cells × 100%. The experiments for the migration, proliferation and the apoptosis assays were performed in triplicate.

### Nitric oxide (NO) and cytokine production analysis

PBMCs (1 × 10^6^ cells/100 μl/well) were seeded into 96-well plates and incubated with r*Eg*ANXB18, r*Eg*ANXB20 (0.5, 1, 2, and 4 μg), pET-32a vector protein (1 μg), concanavalin A (ConA) (1 μg), or 1 μl PBS, respectively, at 37 °C for 48 h. NO production was detected using a Total Nitric Oxide Assay kit (Beyotime).

For cytokine production analysis, PBMCs (1 × 10^6^ cells/ml/well) were seeded into 24-well plates and incubated with r*Eg*ANXB18, r*Eg*ANXB20 (5, 10, 20, and 40 μg), pET-32a vector protein (10 μg), or 10 μl of PBS at 37 °C for 24 h. The specific primers are shown in Additional file 1: Table S3, and qPCR was performed according to the procedure of qPCR method as above with a different annealing temperature at 61 ℃. The *GAPDH* (GenBank BC096440.1) gene from mouse was selected as the housekeeping gene, and the relative expression levels of cytokines were analyzed using the 2^−ΔΔCt^ method.

### Statistical analysis

xStatistical analysis was conducted using GraphPad Prism (version 8.0.2; GraphPad Inc., La Jolla, CA, USA), and data are presented as the mean ± standard deviation (SD). An unpaired Student’s t-test was used for comparisons between two groups, and one-way analysis of variance (ANOVA) was applied to compare differences among three or more groups. *P* < 0.05 indicated statistical significance.

## Results

### Bioinformatic analysis of *Eg*ANXBs

The full-length coding sequences of four *Eg*ANXBs were successfully cloned and sequenced. *Eg*ANXB18, *E*gANXB20, and *Eg*ANXB23 shared the same sequences with their reference sequences, while *EgANXB2* (1065 bp) was 21 bp longer than its reference sequence. The molecular weight of four *Eg*ANXBs ranged from 35.72 kDa to 39.95 kDa. All four *Eg*ANXBs were predicted to be stable, without signal peptides or transmembrane regions (Table 1).

**Table 1:** Bioinformatic analysis of four *Eg*ANXBs

Gene	Amino acid (aa)	Molecular weight (kDa)	PI	Instability index	Signal peptide	Transmembrane area	Dimer formation
*Eg*ANXB2	354	39.95	6.05	35.31	–	–	–
*Eg*ANXB18	348	38.76	4.82	27.03	–	–	–
*Eg*ANXB20	323	36.68	5.17	36.09	–	–	–
*Eg*ANXB23	318	35.72	5.79	36.27	–	–	Yes

**Fig. 1:**
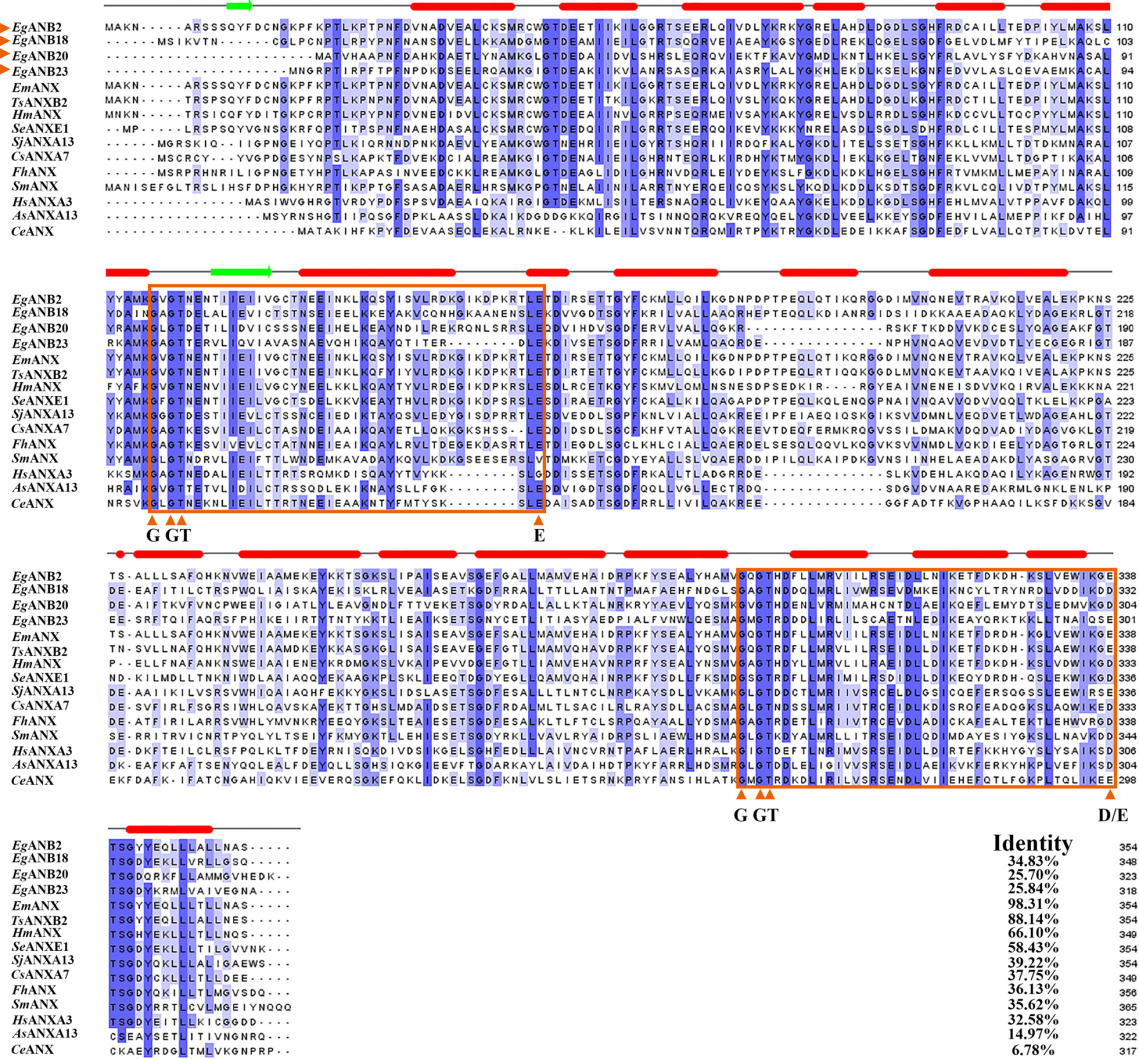
Sequence alignment analysis of *Eg*ANXBs with homologs from other parasites and humans. All the sequences were retrieved from the GenBank and WormBase ParaSite (WP) databases, with the following accession numbers: *Eg*ANXB2, *Echinococcus* *granulosus* (WP: EgrG_000193700); EgANXB18,* E*. *granulosus* (WP: EgrG_000041200); EgANXB20, *E*. *granulosus* (WP: EgrG_000244000); EgANXB23, *E*. *granulosus* (WP: EgrG_000237700); Em, *E*. *multilocularis* (GenBank:CDI98110.1); Ts, *Taenia solium* (GenBank: AAY17503.1); Hm, *Hymenolepis microstoma* (GenBank: CDS30725.1); Se, *Spirometra erinaceieuropaei* (GenBank: ADM26238.1); Sj, *Schistosoma japonicum* (GenBank: CAX70813.1); Cs, *Clonorchis sinensis* (GenBank: GAA48684.1); Fh, *Fasciola hepatica* (GenBank: THD28190.1); Sm, *Schistosoma mansoni* (GenBank: AAC79802.3); Hs, *Homo sapiens* (GenBank:NP_005130.1); As, *Ascaris suum* (GenBank: ADY44710.1); Ce, *Caenorhabditis elegans* (GenBank:CAA83598.1). The identity referred to the identity rate of homologs to EgANXB2. The type II calcium binding sites with the sequence GxGT -[38–40 residues]-D/E are indicated using orange boxes. The α-helix and irregular coil domains are marked with red bars and green arrows, respectively

**Fig. 2:**
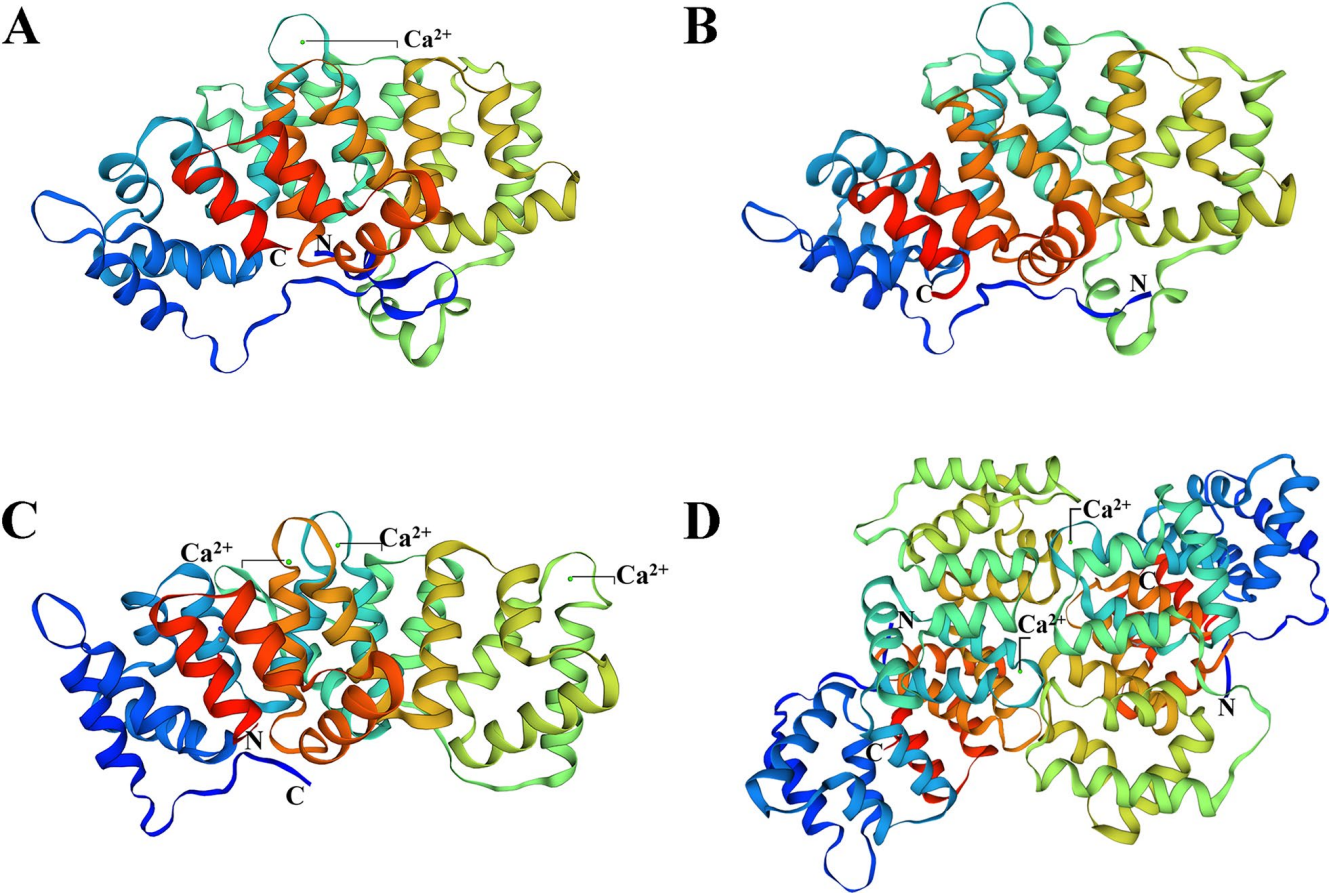
Predicted three-dimensional structures of *Eg*ANXB2 (**A**), *Eg*ANXB18 (**B**), *Eg*ANXB20 (**C**), and *Eg*ANXB23 (**D**). C, C terminal; N, N terminal. The Ca-binding sites were indicated, and *Eg*ANXB23 was predicted to form a dimer

**Fig. 3:**
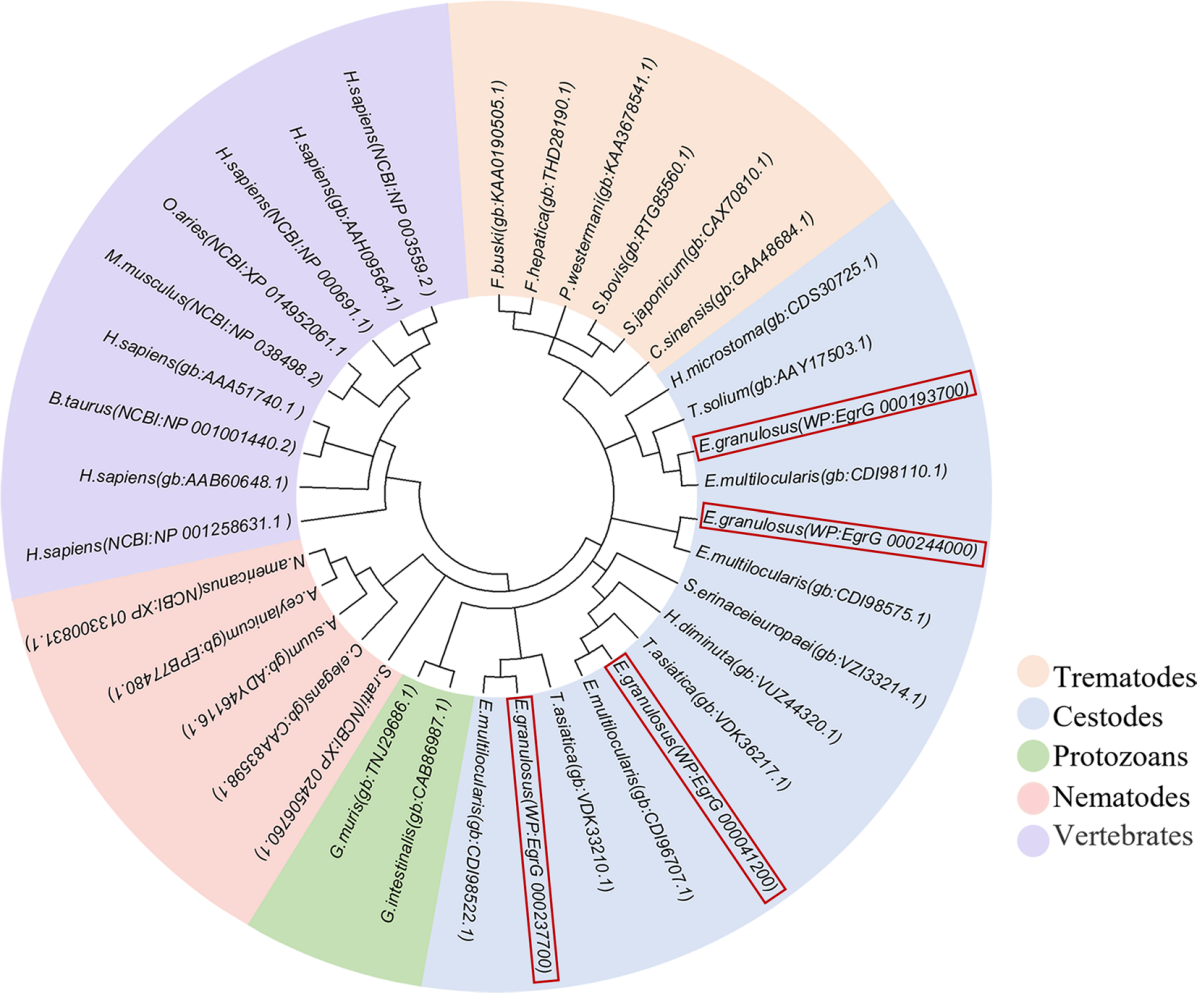
Phylogenetic tree of four *Eg*ANXBs (in red boxes) based on maximum likelihood statistical method. All the sequences were retrieved from the GenBank and WormBase ParaSite (WP) databases, with the following accession numbers: *Eg*ANXB2, *Echinococcus* *granulosus* (WP: EgrG_000193700); EgANXB18,* E*. *granulosus* (WP: EgrG_000041200); EgANXB20, *E*. *granulosus* (WP: EgrG_000244000); EgANXB23, *E*. *granulosus* (WP: EgrG_000237700). WP, WormBase ParaSite; gb, GenBank

Multiple sequence alignment analysis showed that *Eg*ANXB2 had relatively low identity with *Eg*ANXB18, *Eg*ANXB20, or *Eg*ANXB23, and these four *Eg*ANXBs exhibited > 97% identity with *Echinococcus multilocularis* orthologs (Fig. 1). Moreover, all four *Eg*ANXBs contained two type II calcium binding sites with the GxGT- {38–40 amino acid residues}-D/E sequence, in accordance with the canonical characteristics of the annexin superfamily [30]. The *Eg*ANXBs mainly contained α-helices, followed by random coils, β-turns, and β-folds, which was further confirmed by three-dimensional structure prediction (Fig. 2). The four *Eg*ANXBs were predicted to form an overall shape of a slightly bent disc, including a convex side and a concave side. The type II calcium binding sites were located on the convex side, except in *Eg*ANXB18, which was predicted to lack a calcium binding site. In addition, *Eg*ANXB23 was predicted to form dimers. The maximum likelihood phylogenetic tree showed that the four *Eg*ANXBs were located in different branches, exhibiting the closest evolutionary relationships with *E. multilocularis* annexins (Fig. 3).

### Expression, purification, and western blotting of r*Eg*ANXBs

**Fig. 4:**
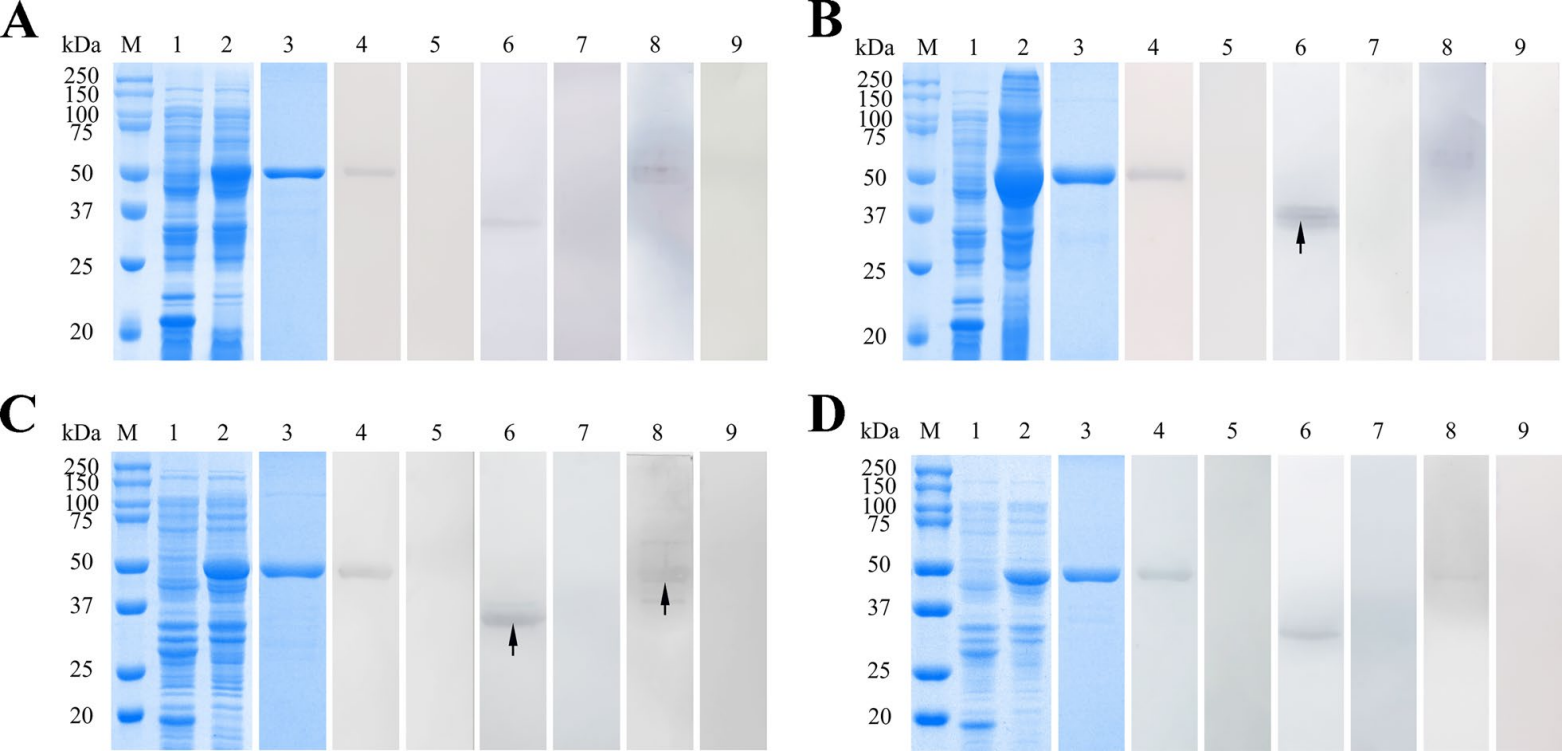
SDS-PAGE and western blotting analysis of the purified r*Eg*ANXB2 (**A**), r*Eg*ANXB18 (**B**), r*Eg*ANXB20 (**C**), and r*Eg*ANXB23 (**D**). M, protein molecular weight marker; 1, pET32a( +)-vector proteins induced by IPTG; 2, proteins of pET32a( +)-*Eg*ANXBs induced by IPTG; 3, purified r*Eg*ANXBs; 4, western blotting of purified r*Eg*ANXBs probed with anti-r*Eg*ANXBs rat IgG; 5, western blotting of purified r*Eg*ANXBs probed with negative rat serum; 6, western blotting of extracted total protein of PSCs probed with anti-r*Eg*ANXBs rat IgG;7, western blotting of extracted total protein of PSCs probed with negative rat serum; 8, western blotting of purified r*Eg*ANXBs probed with serum from *E*. *granulosus* infected sheep; 9, western blotting of purified r*Eg*ANXBs probed with serum from *Echinococcus* *granulosus* non-infected sheep

**Fig. 5:**
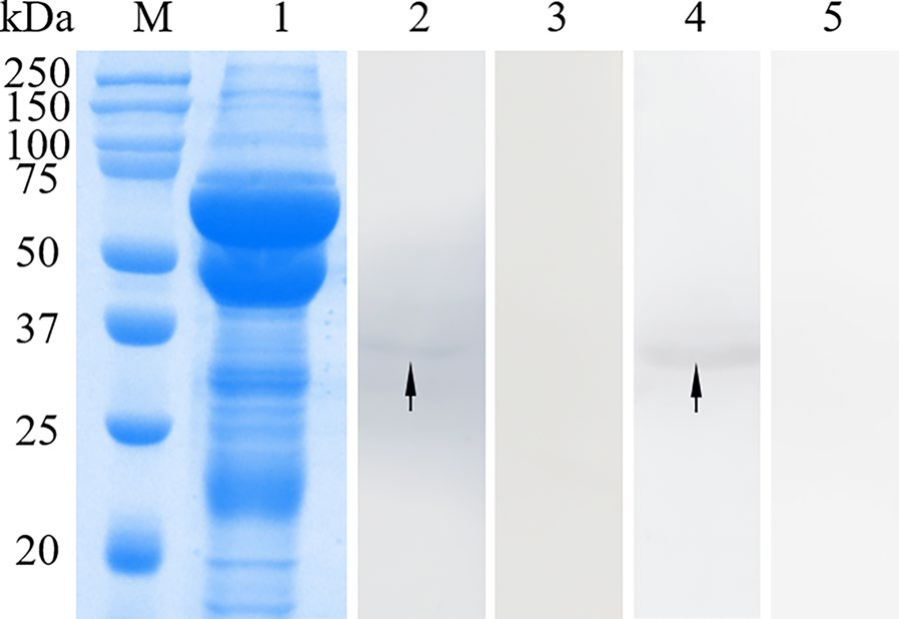
SDS-PAGE and western blotting analysis of cyst fluid. M, protein molecular weight marker; 1, SDS-PAGE of total proteins within cyst fluid; 2, total proteins within cyst fluid reacted with anti-r*Eg*ANXB18 rat IgG; 3 and 5, total proteins within cyst fluid reacted with negative rat serum; 4, total proteins within cyst fluid reacted with anti-r*Eg*ANXB20 rat IgG

The r*Eg*ANXB2, r*Eg*ANXB18, r*Eg*ANXB20, and r*Eg*ANXB23 were successfully expressed in a soluble form with expected molecular weights of approximately 57 kDa, 56 kDa, 54 kDa, and 53 kDa, respectively (Fig. 4). Western blotting demonstrated that the r*Eg*ANXBs could be specifically recognized by their corresponding anti-r*Eg*ANXBs rat IgG and positive sera from naturally infected sheep (Fig. 4: Lanes 4, 8), while no signal was detected in the presence of negative sera (Fig. 4: Lanes 5, 9). Moreover, the anti-r*Eg*ANXBs rat IgG was able to recognize the four *Eg*ANXBs among the extracted total proteins from PSCs (Fig. 4: Lane 6). Furthermore, *Eg*ANXB18 and *Eg*ANXB20 were detected in cyst fluid (Fig. 5: Lane 2, 4).

### Ca^2+^-dependent phospholipid binding bioactivity of r*Eg*ANXBs

**Fig. 6:**
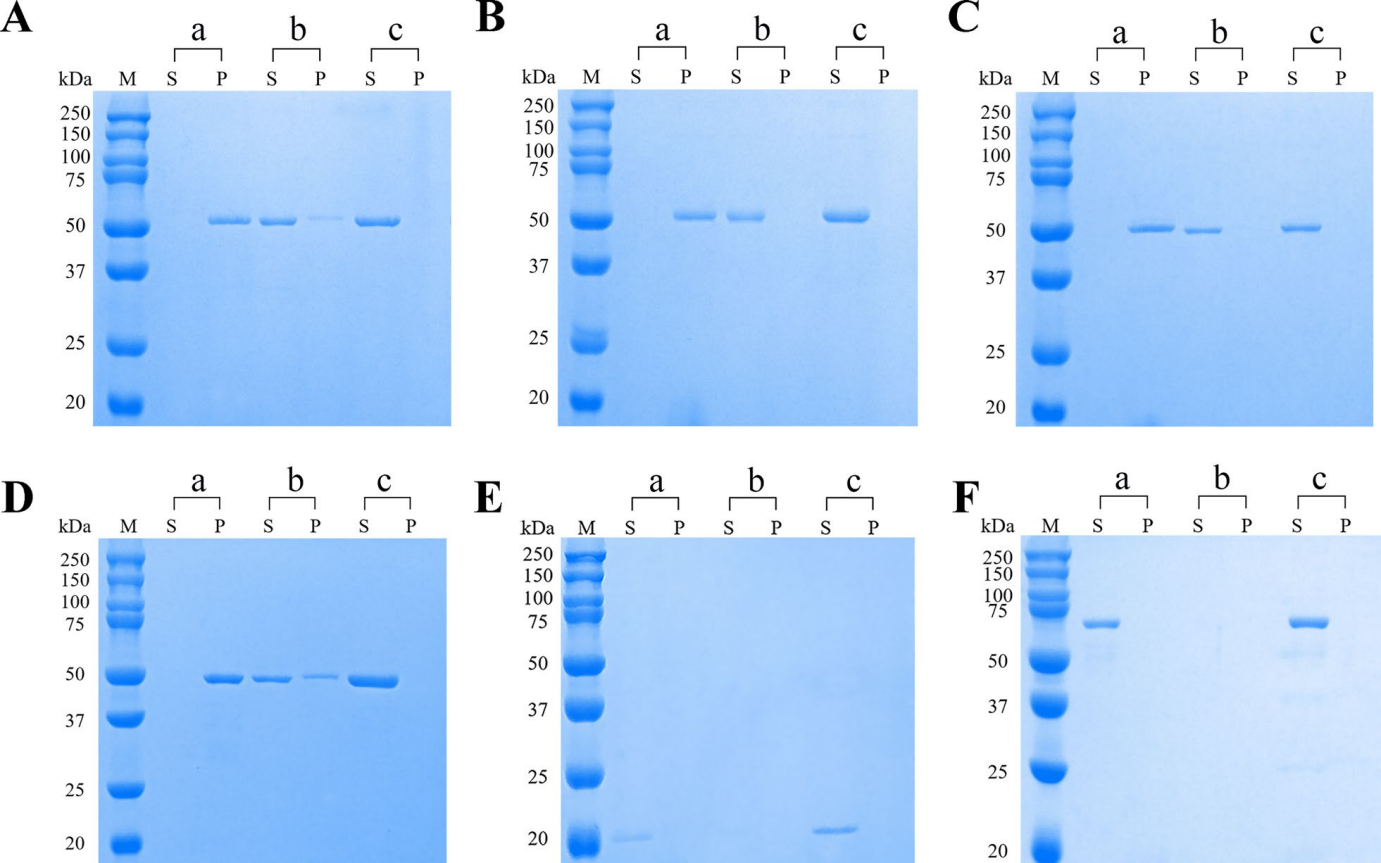
Phospholipid-binding bioactivity in Ca^2+^-dependent manner of r*Eg*ANXB2 (**A**), r*Eg*ANXB18 (**B**), r*Eg*ANXB20 (**C**), r*Eg*ANXB23 (**D**), pET32a vector expression protein (E), and BSA (F). Group a and b contained the mixture of protein, liposomes, CaCl_2_, and Tris–HCl, while group c excluded CaCl_2_. M, protein molecular weight marker; S, supernatant; P, precipitate

Our findings showed that all four r*Eg*ANXBs were able to bind to liposomes in the presence of Ca^2+^ and remained in the precipitate after centrifugation (Fig. 6: a group), while they could not bind to liposomes in the absence of Ca^2+^ and thus remained in the supernatant (Fig. 6: c group). Meanwhile, as EDTA was added to competitively bind Ca^2+^, the r*Eg*ANXBs were released from liposomes and reappeared in the supernatant (Fig. 6: b group). Notably, r*Eg*ANXB2 and r*Eg*ANXB23 were not completely released from liposomes after EDTA addition, and some of them remained in the precipitate (Fig. 6A, D). In addition, pET 32a vector expression protein and BSA were analyzed, and no Ca^2+^-dependent phospholipid binding characteristics were observed (Fig. 6E, F), suggesting that the Ca^2+^-dependent phospholipid binding property was solely because of r*Eg*ANXBs rather than the pET-32a vector expression protein or other factors.

### Localization of *Eg*ANXBs by immunofluorescence assays

**Fig. 7:**
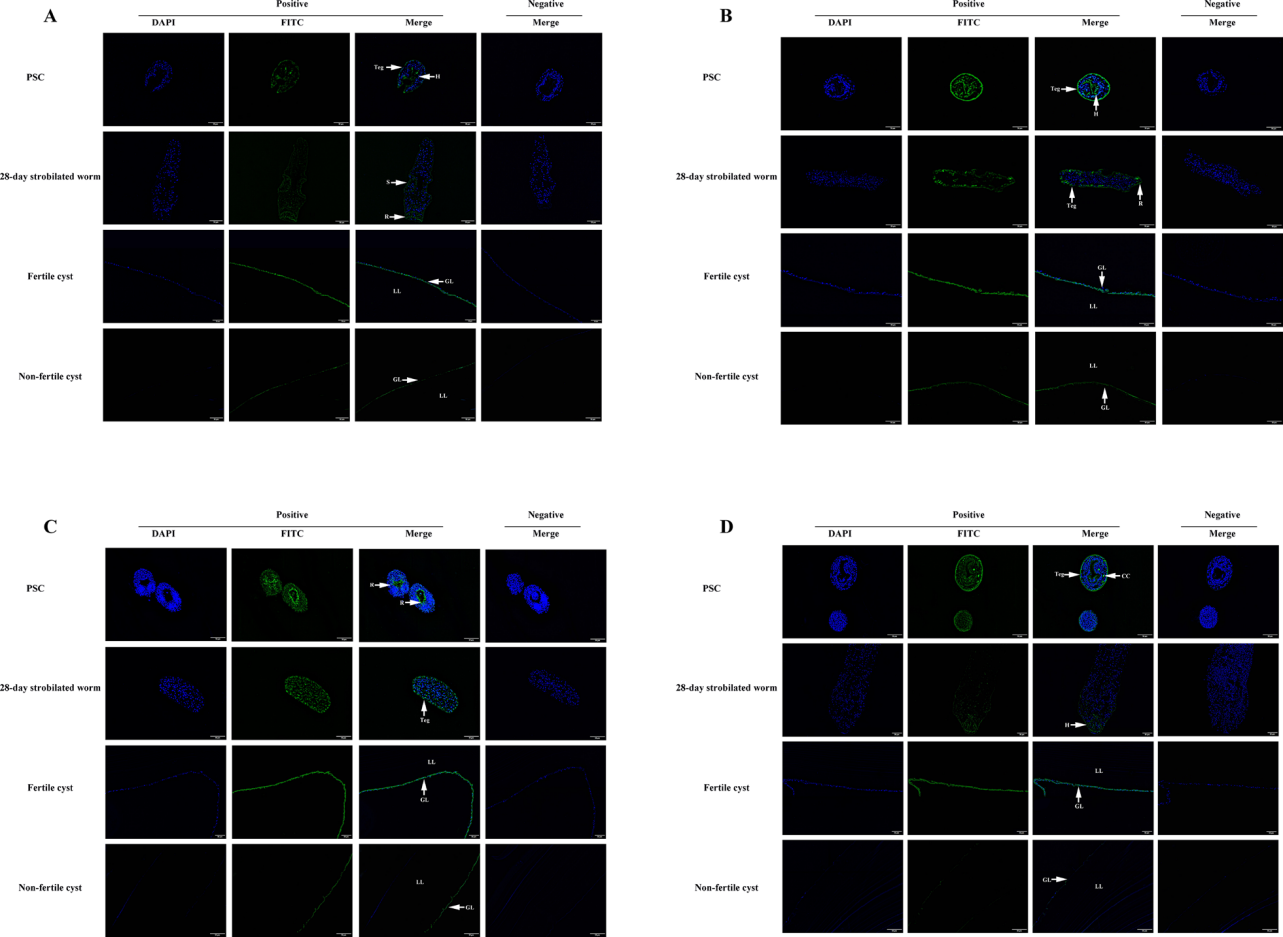
Immunolocalization of *Eg*ANXB2 (**A**), *Eg*ANXB18 (**B**), *Eg*ANXB20 (**C**), and *Eg*ANXB23 (**D**) in different development stages and fertile/ non-fertile cysts of *Echinococcus granulosus*. Teg, tegument; H, hooks; R, rostellum; S, sucker; GL, germinal layer; LL, laminated layer; CC, calcareous corpuscles

Immunolocalization analysis showed that both *Eg*ANXB2 and *Eg*ANXB18 were mainly distributed in the tegument and hooks of PSCs (Fig. 7A, B), while *Eg*ANXB20 was predominantly localized in the rostellum of PSCs (Fig. 7C). *Eg*ANXB23 was mainly distributed in the tegument and calcareous corpuscles of PSCs (Fig. 7D). In 28-day strobilated worms, *Eg*ANXB2 and *Eg*ANXB20 were widely distributed, while *Eg*ANXB18 was mainly distributed in the tegument and rostellum, and *Eg*ANXB23 was only distributed in hooks. Interestingly, all four *Eg*ANXBs were found to be similarly distributed in the germinal layer of fertile cysts, with less distribution in the germinal layer of non-fertile cysts. The *Eg*ANXB23 showed almost undetectable fluorescence signals.

### Transcription levels of *Eg*ANXBs in PSCs and 28-day strobilated worms

**Fig. 8:**
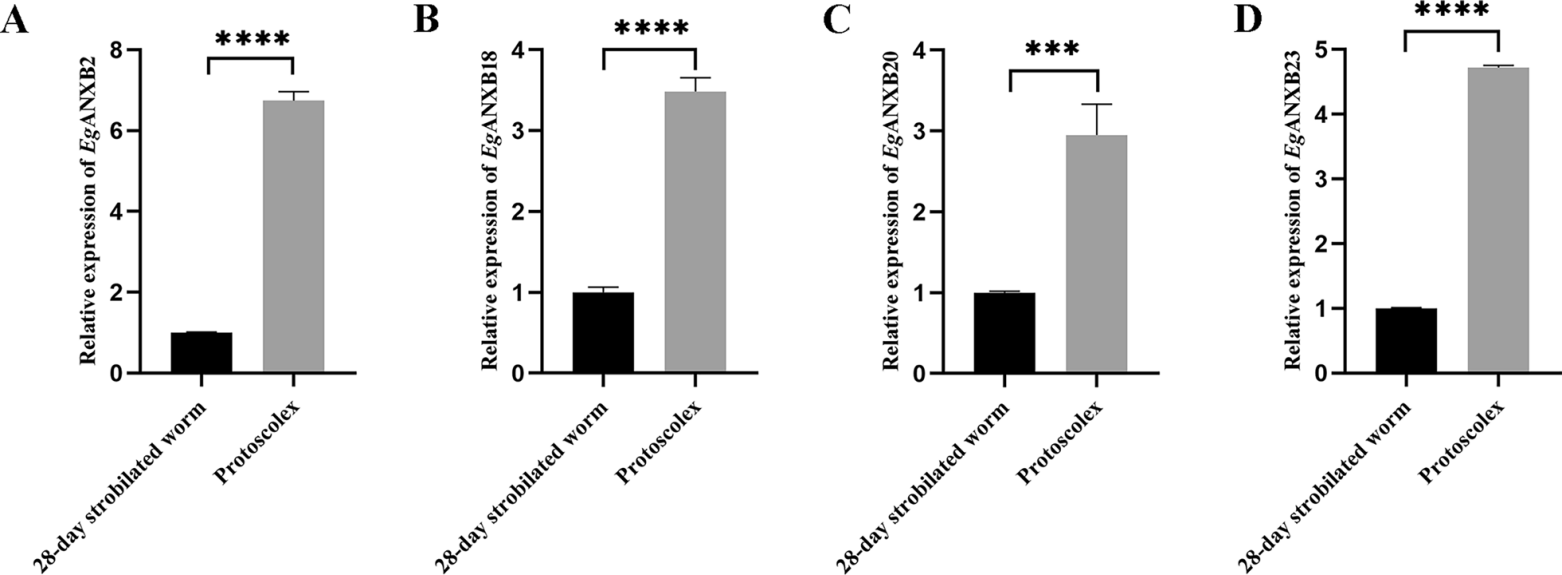
Transcription levels of *Eg*ANXB2 (**A**), *Eg*ANXB18 (**B**), *Eg*ANXB20 (**C**), and *Eg*ANXB23 (**D**) in protoscoleces and 28-day strobilated worms. Data are displayed as the mean ± SD, ***P < 0.001, ****P < 0.0001 (Student’s t-tests)

The qRT-PCR analysis showed that four *Eg*ANXBs were transcribed in both the PSCs and 28-day strobilated worms, and the transcription levels of *Eg*ANXB2, *Eg*ANXB18, *Eg*ANXB20, and *Eg*ANXB23 in PSCs were significantly higher than those in the 28-day strobilated worms (Fig. 8).

### r*Eg*ANXBs bind to PBMCs by immunofluorescence assays

**Fig. 9:**
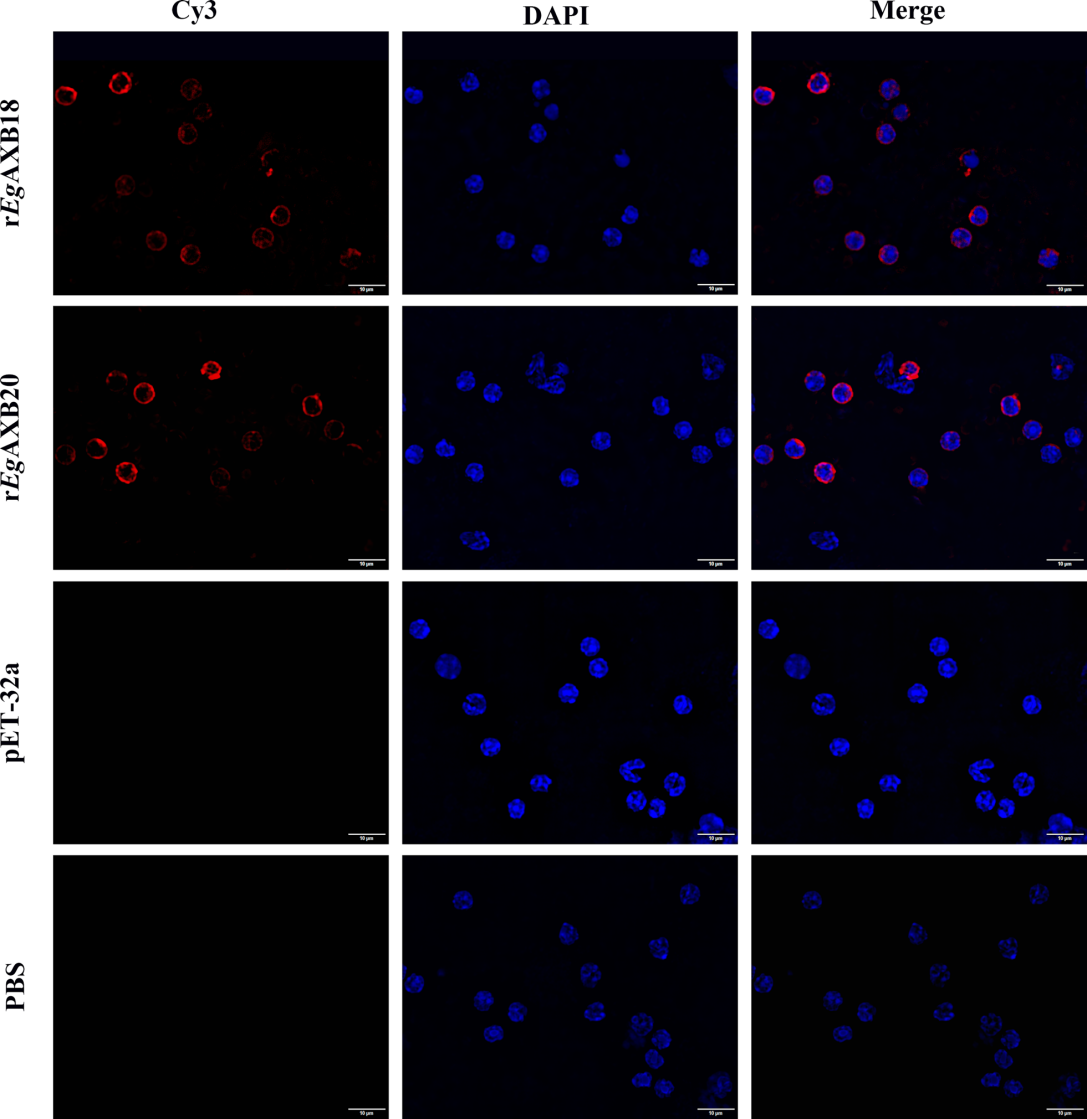
r*Eg*ANXBs bind to mouse PBMCs, as identified by immunofluorescence staining. The r*Eg*ANXBs were used as the primary antibody, and DAPI, Cy3 and PBS referred to staining, secondary antibody, and control without primary antibody, respectively

When PBMCs were incubated with r*Eg*ANXB18 or r*Eg*ANXB20, red fluorescence was observed on the cell surface (Fig. 9), while the PBMCs incubated with pET-32a vector expression protein or PBS showed no red fluorescence, suggesting that r*Eg*ANXB18 and r*Eg*ANXB20 could bind to PBMCs.

### Effects of r*Eg*ANXBs on PBMC proliferation, apoptosis, and migration

**Fig. 10:**
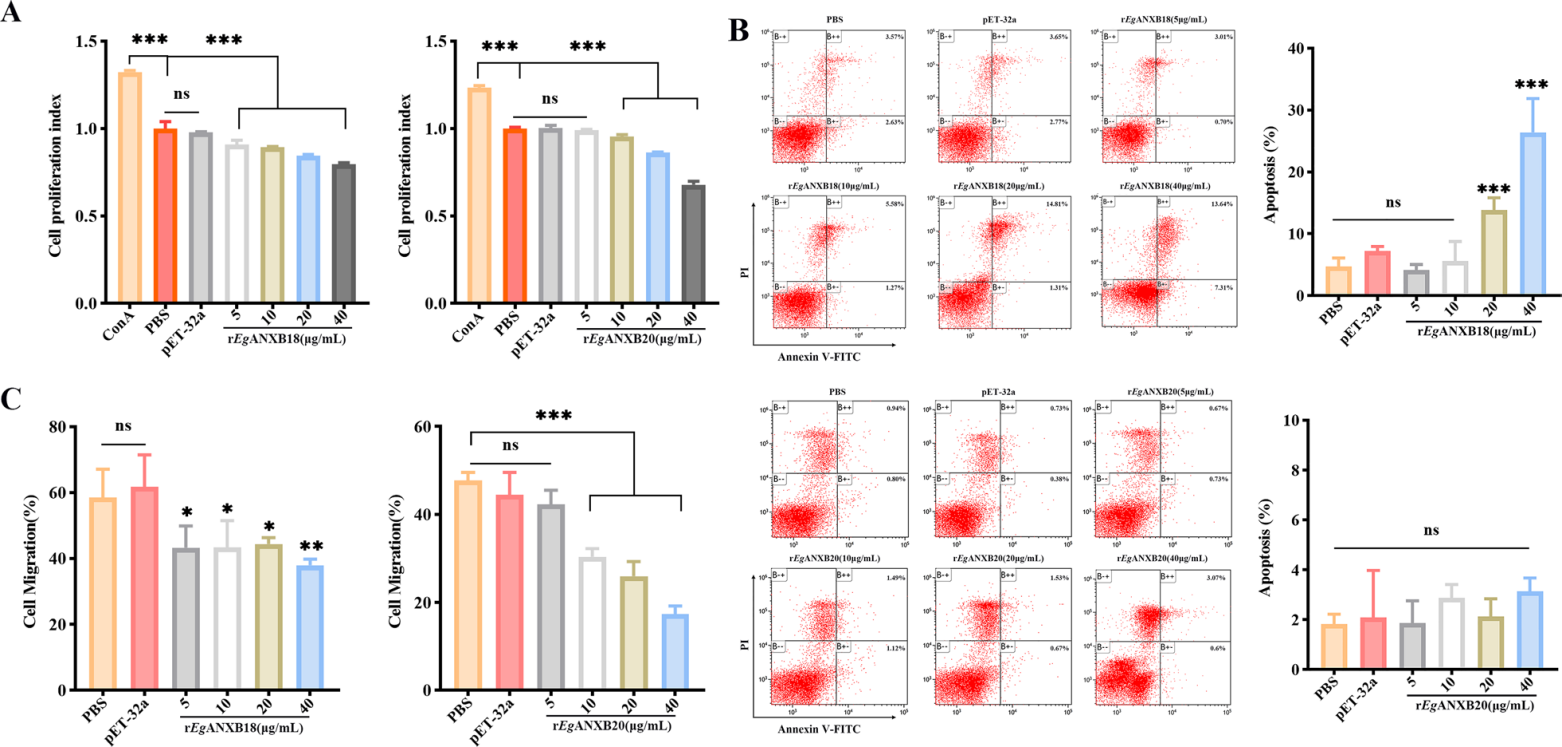
Effects of r*Eg*ANX18 and r*Eg*ANX20 on mouse PBMCs proliferation (**A**), apoptosis (**B**), and migration (**C**). Statistical analysis between the PBS and other groups was conducted using one-way ANOVA with data displayed as the mean ± SD, *P < 0.05, **P < 0.01, ***P < 0.001; ns: not significant. ANOVA, analysis of variance

The CCK-8 assay showed that r*Eg*ANXB18 inhibited PBMC proliferation in a dose-dependent manner (Fig. 10A). Additionally, we observed that a low concentration (5 μg/ml) of r*Eg*ANXB20 had no effect on PBMC proliferation, while high concentrations of r*Eg*ANXB20 significantly inhibited it (*P* < 0.001). As a positive control, ConA could induce the proliferation of PBMCs (*P* < 0.001). Moreover, we investigated the effect of r*Eg*ANXB18 and r*Eg*ANXB20 on PBMC apoptosis. Our data demonstrated that r*Eg*ANXB18, at high concentrations (20 μg/ml and 40 μg/ml), significantly promoted PBMC apoptosis (*P* < 0.001). However, r*Eg*ANXB20 showed no effects on PBMC apoptosis (Fig. 10B). To assess the influence of r*Eg*ANXB18 and r*Eg*ANXB20 on PBMC migration, we conducted a Transwell migration assay. The results showed that both r*Eg*ANXB18 (*P* < 0.05) and r*Eg*ANXB20 (*p* < 0.001; except for 5 μg/ml) inhibited PBMC migration (Fig. 10C).

### Effects of r*Eg*ANXBs on NO production and expression of cytokines in PBMCs

**Fig. 11:**
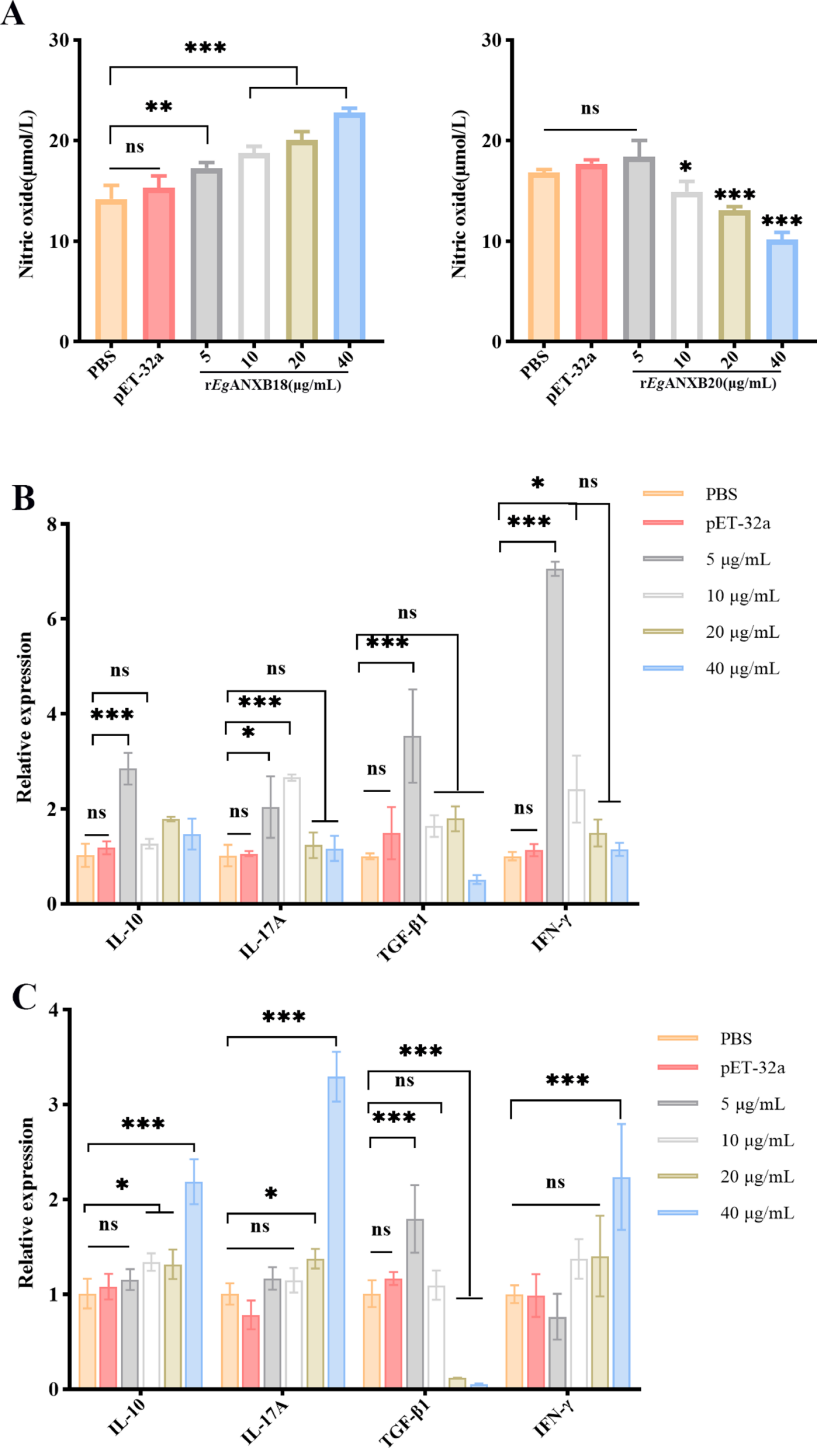
Effects of r*Eg*ANX18 and r*Eg*ANX20 on mouse PBMCs nitric oxide production (**A**) and cytokines expression (**B**: r*Eg*ANX18, **C**: r*Eg*ANX20). Statistical analysis was performed using one-way ANOVA, and *P* values were indicated as follows: *P < 0.05, **P < 0.01, ***P < 0.001; ns: not significant. IL-10, interleukin 10; IL-17, interleukin 17; TGF-β1, transforming growth factor beta 1; IFN-γ, interferon gamma

To investigate the effect of r*Eg*ANXBs on the production of NO by PBMCs, we measured NO in the cell culture supernatant. The results demonstrated that r*Eg*ANXB18 and r*Eg*ANXB20 had completely different effects on NO production. Specifically, r*Eg*ANXB18 induced NO production in a dose-dependent manner (*P* < 0.05), while r*Eg*ANXB20(10, 20, and 40 μg/ml) inhibited it (*P* < 0.05) (Fig. 11A). Meanwhile, qRT-PCR analysis showed that low concentrations of r*Eg*ANXB18 (5 μg/ml) could markedly promote the relative mRNA expression levels of cytokines interleukin (IL)-10, IL-17A, transforming growth factor beta 1 (TGF-β1), and interferon gamma (IFN-γ) (*P* < 0.05). However, with increasing recombinant protein concentration, the expression levels of the cytokines showed no significance compared to those in the PBS group (Fig. 11B). By contrast, r*Eg*ANXB20 induced the expression of IL-10, IL-17A, and IFN-γ in a dose-dependent manner. In addition, 5 μg/ml r*Eg*ANXB20 induced the expression of TGF-β1 (*P* < 0.001) while 20 μg/ml and 40 μg/ml r*Eg*ANXB20 inhibited its expression (Fig. 11C).

## Discussion

The metacestode of *E. granulosus* survives within its hosts, producing ESPs that are generally considered to play essential roles in parasite invasion, immune escape, and metabolic adaptation [5]. Some annexins from *Taenia* *solium*, as secretory proteins discovered in ESPs, were believed to be involved in modulating host immune responses and facilitating parasite immune evasion [31]. However, the functional characteristics of *E*. *granulosus* annexins have yet to be clearly determined. In this study, we aimed to expand the current understanding of annexins by characterizing four novel *Eg*ANXBs and exploring their potential roles in parasite immune evasion. Annexins belong to an evolutionarily conserved protein superfamily characterized by their ability to reversibly interact with membranes in a calcium-dependent manner [30].

Annexins are composed of a C- and an N-terminal domain. The C-terminal domain contains four or eight repeats arranged to form five α helices that finally form the overall shape of a slightly curved disc with a central hydrophilic hole [32, 33]. Herein, we observed that all four *Eg*ANXBs contained the sequence GXGT- {38–40 amino acid residues}-D/E and shared similar conventional three-dimensional structures. Notably, although the GXGT- {38–40 amino acid residues}-D/E sequence was present in *Eg*ANXB18, no calcium-binding site was predicted in the protein's three-dimensional structure. Since *Eg*ANXB18 was experimentally shown to bind phospholipids in a Ca^2+^-dependent manner, this unexpected outcome could be attributed to the template selected to construct the model. *Eg*ANXB23 was predicted to form a dimer, which is consistent with previous reports indicating that certain annexins, such as ANXA2 and ANXA5, could form dimers and trimers, and even heterotetramers, with other molecules, such as the S100 protein [34–36].

The four r*Eg*ANXBs were confirmed to bind to phospholipids in a Ca^2+^-dependent manner; intriguingly, although r*Eg*ANXB2 and r*Eg*ANXB23 could bind to phospholipids, they were not completely released when EDTA was added, likely because of the formation of transmembrane ion channels. Previous studies have shown that several annexins, including ANXA1, ANXA2, ANXA5, ANXA6, and ANXA7 of humans and other species, exhibit transmembrane ion channel activity [37, 38]. Normally, most of the annexins could detach from the membrane in the absence of Ca^2+^. However, transmembrane ion channels would keep annexins bound to the membrane, and it was observed that ANXA1 and ANXA2 were resistant to EDTA extraction and required detergent treatment for the release from the membrane [39, 40]. Although more data are needed, we postulated that r*Eg*ANXB2 and r*Eg*ANXB23 from *E. granulosus* possibly attach to the membranes and then insert into them to form transmembrane ion channels, such that EDTA failed to extract them from the liposomes.

The distribution of four *Eg*ANXBs in different development stages of *E. granulosus* differed from each other, which was also observed in trematodes [41–44]. Surprisingly, higher transcription levels of the four *Eg*ANXBs were detected in PSCs compared with those in 28-day strobilated worms, which contrasted with *Eg*ANXB3 and *Eg*ANXB38 [20]. This suggested a more intimate relationship between *Eg*ANXBs in this study and PSCs. Furthermore, *Eg*ANXB18 and *Eg*ANXB20 were identified as secretory proteins despite lacking signal peptides, suggesting that they were probably secreted through unconventional secretion pathways, such as direct translocation across the plasma membrane or secreted via extracellular vesicles [45]. Under the assayed conditions, *Eg*ANXB2 and *Eg*ANXB23 were not detected in cyst fluid, indicating that they were not secretory proteins, or that their concentrations were too low.

Annexins are generally considered to function intracellularly [46]. However, several secretory annexins have been identified to be involved in parasite-host interactions including *Clonorchis sinensis* annexin B30 [41], *T. solium* annexin B1 [31], and *E. granulosus* annexins B3, B33, and B38 [20, 21]. Furthermore, previous evidence has shown that secretory annexins could bind to immune cells via specific receptors, such as formylated peptide receptors (FPR or ALXR) and CD44 of neutrophils for ANXA1 and ANXA2, respectively [47–49]. Similarly, both r*Eg*ANXB18 and r*Eg*ANXB20 were observed to bind to PBMCs and inhibit their proliferation, and high concentrations of r*Eg*ANXB18 also promoted PBMC apoptosis, while r*Eg*ANXB20 showed no effect. This suggested that r*Eg*ANXB18 and r*Eg*ANXB20 were more likely to suppress the immune response by affecting proliferation rather than apoptosis.

Immune cells can be recruited and migrate to specific tissues when an inflammatory response occurs, which is essential for infection control, tissue repair, and immune surveillance [50]. Several factors, including annexins, are involved in the modulation of cell migration. For instance, ANXA1 inhibits the activation of integrins induced by CCL5, CCL2, or CXCL1 [51], thereby affecting the migration and aggregation of leukocytes and suppressing the adhesion and migration of neutrophils to vascular endothelial cells [47]. In this study, we observed that both r*Eg*ANXB18 and r*Eg*ANXB20 inhibited the migration of PBMCs, implying that they potentially played a role in the modulation of the immune response in hosts.

NO, a multifunctional bioactive molecule, is generally considered to play an important role in anti-parasite infection by inhibiting cellular energy synthesis or destroying proteins, lipids, and nucleic acids [52]. Our study showed that r*Eg*ANXB18 and r*Eg*ANXB20 exhibited opposite effects on NO production. Specifically, r*Eg*ANXB18 promoted NO production, while r*Eg*ANXB20 inhibited it, suggesting that both proteins affected NO production, probably by regulating the expression or activity of inducible nitric oxide synthases (iNOS). Previous studies confirmed that iNOS transcription was induced by IFN-γ and lipolysaccharide (LPS) [53]. We observed that high concentrations of EgANXB18 do not induce significant IFN-γ production but do induce NO production. In contrast, since r*Eg*ANXB20 affected the expression of IFN-γ only at the highest concentration, it is likely to function through a different pathway.

Th1 and Th2 immune responses coexist in patients with CE, with Th1 cytokines playing a role in parasite removal and Th2 cytokines being linked to parasite immune evasion [54]. IFN-γ, a Th1 cytokine, has been proven to enhance the activity and antigen-presenting ability of Th1 cells and macrophages while inhibiting the differentiation and function of Tregs and Th2 and Th17 cells [55]. Th17/Treg cells are believed to be involved in CE development, with Th17 cells mediating chronic inflammatory responses by secreting IL-17A and Treg cells exerting immunosuppressive effects by producing IL-10 and TGF-β1 [56, 57]. Our findings demonstrated that low concentrations of r*Eg*ANXB18 significantly increased the levels of IL-10, TGF-β1, IL-17A, and IFN-γ mRNA, with the increase of IFN-γ mRNA being markedly higher than those the others. Previous studies have shown that when PSCs were co-cultured PBMCs from CE patients, the addition of IFN-γ promoted the iNOS and NO production, leading to higher PSC mortality [58]. Therefore, it is possible that r*Eg*ANXB18 participates in anti-parasite infection by inducing IFN-γ expression. In addition, r*Eg*ANXB20 promoted the expression of IL-10, IL-17A, and IFN-γ. Moreover, it also significantly increased the expression level of TGF-β1 at low concentrations while showing opposite effects at high concentrations. Taken together, r*Eg*ANXB20 induced the expression of both Th1/Th2 and Th17/Treg cytokines, consistent with the cytokine profile observed in patients with CE.

## Conclusion

The present study identified four *Eg*ANXBs as typical annexins with canonical structures and Ca^2+^-dependent phospholipid binding properties. Notably, *Eg*ANXB2 and *Eg*ANXB23 were postulated to have ion channel activities when interacting with membranes. Immunolocalization and qRT-PCR analysis revealed that both the distribution and transcription levels of these four *Eg*ANXBs in PSCs were higher than in 28-day strobilated worms, indicating their involvement in the growth and development of PSCs. Furthermore, *Eg*ANXB18 and *Eg*ANXB20 were found to be secretory proteins that could bind to PBMCs to regulate cell proliferation, apoptosis, migration, and NO and cytokine production. These results suggest potential roles of *Eg*ANXB18 and *Eg*ANXB20 in immune evasion strategies employed by the parasites.

### Supplementary Information

**Additional file 1: Table S1. **Primers for the PCR amplification of four *Eg*ANXBs. **Table S2. **Primers for the qRT-PCR amplification of four *Eg*ANXBs and *GAPDH*. **Table S3.** Primers for the qRT-PCR of cytokines.

## Data Availability

All data in this study are included in the article material. Any inquiries can be directed to the corresponding author.

## Abbreviations

*E*. *granulosus* s.l.: *Echinococcus granulosus* Sensu lato
CE: Cystic echinococcosis
PSCs: Protoscoleces
rEgANXBs: Recombinant *Echinococcus granulosus* annexin B proteins

## References

[1] Deplazes P, Rinaldi L, Rojas CA, Torgerson P, Harandi M, Romig T (2017). Global distribution of alveolar and cystic echinococcosis. Adv Parasitol.

[2] Periago MV (2023). Special issue *Echinococcosis*. Parasitologia..

[3] Woolsey ID, Miller AL (2021). *Echinococcus granulosus* sensu lato and *Echinococcus multilocularis*: a review. Res Vet Sci.

[4] Wen H, Vuitton L, Tuxun T, Li J, Vuitton DA, Zhang W (2019). Echinococcosis: advances in the 21st century. Clin Microbiol Rev.

[5] Pan W, Shen Y, Han X, Wang Y, Liu H, Jiang Y (2014). Transcriptome profiles of the protoscoleces of *Echinococcus granulosus* reveal that excretory-secretory products are essential to metabolic adaptation. PLoS Negl Trop Dis.

[6] Virginio VG, Taroco L, Ramos AL, Ferreira AM, Zaha A, Ferreira HB (2007). Effects of protoscoleces and AgB from *Echinococcus granulosus* on human neutrophils: possible implications on the parasite’s immune evasion mechanisms. J Parasitol Res.

[7] Rigano R, Buttari B, Profumo E, Ortona E, Delunardo F, Margutti P (2007). *Echinococcus granulosus* antigen B impairs human dendritic cell differentiation and polarizes immature dendritic cell maturation towards a Th2 cell response. Infect Immun.

[8] Wang H, Zhang C, Fang B, Li Z, Li L, Bi X (2019). Thioredoxin peroxidase secreted by *Echinococcus granulosus* (sensu stricto) promotes the alternative activation of macrophages via PI3K/AKT/mTOR pathway. Parasit Vectors.

[9] Rashidi S, Mansouri R, Ali-Hassanzadeh M, Muro A, Nguewa P, Manzano-Román R (2023). The most prominent modulated annexins during parasitic infections. Acta Trop.

[10] Boye TL, Maeda K, Pezeshkian W, Sønder SL, Haeger SC, Gerke V (2017). Annexin A4 and A6 induce membrane curvature and constriction during cell membrane repair. Nat Commun.

[11] Gabel M, Delavoie F, Demais V, Royer C, Bailly Y, Vitale N (2015). Annexin A2-dependent actin bundling promotes secretory granule docking to the plasma membrane and exocytosis. J Cell Biol.

[12] Benaud C, Le Dez G, Mironov S, Galli F, Reboutier D, Prigent C (2015). Annexin A2 is required for the early steps of cytokinesis. EMBO Rep.

[13] Huang Y, Du Y, Zhang X, Bai L, Mibrahim M, Zhang J (2015). Down-regulated expression of Annexin A7 induces apoptosis in mouse hepatocarcinoma cell line by the intrinsic mitochondrial pathway. Biomed Pharmacother.

[14] Gobbetti T, Cooray SN (2016). Annexin A1 and resolution of inflammation: tissue repairing properties and signalling signature. J Biol Chem.

[15] Cai M, Yang J, Li Y, Ding J, Kandil OM, Kutyrev I (2021). Comparative analysis of different extracellular vesicles secreted by *Echinococcus granulosus* protoscoleces. Acta Trop.

[16] Yang J, Wu J, Fu Y, Yan L, Li Y, Guo X (2021). Identification of different extracellular vesicles in the hydatid fluid of *Echinococcus granulosus* and immunomodulatory effects of 110 K EVs on sheep PBMCs. Front Immunol..

[17] Virginio VG, Monteiro KM, Drumond F, de Carvalho MO, Vargas DM, Zaha A (2012). Excretory/secretory products from in vitro-cultured *Echinococcus granulosus* protoscoleces. Mol Biochem Parasitol.

[18] Siles-Lucas M, Sánchez-Ovejero C, González Sánchez M, González E, Falcón-Pérez JM, Boufana B (2017). Isolation and characterization of exosomes derived from fertile sheep hydatid cysts. Vet Parasitol.

[19] Zeghir-Bouteldja R, Polomé A, Bousbata S, Touil-Boukoffa C (2017). Comparative proteome profiling of hydatid fluid from Algerian patients reveals cyst location-related variation in *Echinococcus granulosus*. Acta Trop.

[20] Song H, He X, Du X, Hua R, Xu J, He R (2021). Molecular characterization and expression analysis of annexin B3 and B38 as secretory proteins in *Echinococcus granulosus*. Parasit Vectors.

[21] Song X, Hu D, Zhong X, Wang N, Gu X, Wang T (2016). Characterization of a secretory annexin in *Echinococcus granulosus*. Am J Trop Med Hyg.

[22] Khan A, El-Buni A, Ali M (2001). Fertility of the cysts of Echinococcus granulosus in domestic herbivores from Benghazi, Libya, and the reactivity of antigens produced from them. Ann Trop Med Parasitol.

[23] Zhan J, Song H, Wang N, Guo C, Shen N, Hua R (2020). Molecular and functional characterization of inhibitor of apoptosis proteins (IAP, BIRP) in *Echinococcus granulosus*. Front Microbiol.

[24] Waterhouse AM, Procter JB, Martin DM, Clamp M, Barton GJ (2009). Jalview Version 2—a multiple sequence alignment editor and analysis workbench. Bioinformatics.

[25] Kumar S, Stecher G, Tamura K (2016). MEGA7: molecular evolutionary genetics analysis version 7.0 for bigger datasets. Mol Biol Evol..

[26] Tsai IJ, Zarowiecki M, Holroyd N, Garciarrubio A, Sanchez Flores A, Brooks KL (2013). The genomes of four tapeworm species reveal adaptations to parasitism. Nature.

[27] Li Y, Zhao X, Li M, Yang C, Wang L, Lin J (2013). Fast preparation of a polyclonal antibody against chicken protocadherin. Genet Mol Res.

[28] Livak KJ, Schmittgen TD (2001). Analysis of relative gene expression data using real-time quantitative PCR and the 2− ΔΔCT method. Methods..

[29] Trindade SC, Olczak T, Gomes-Filho IS, Moura-Costa LF, Vale VL, Galdino-Neto M (2012). Porphyromonas gingivalis antigens differently participate in the proliferation and cell death of human PBMC. Arch Oral Biol.

[30] Gerke V, Moss SE (2002). Annexins: from structure to function. Physiol Rev.

[31] Yan H, Xue G, Mei Q, Ding F, Wang Y, Sun S (2008). Calcium-dependent proapoptotic effect of *Taenia solium* metacestodes annexin B1 on human eosinophils: a novel strategy to prevent host immune response. Int J Biochem Cell Biol.

[32] Gerke V, Creutz CE, Moss SE (2005). Annexins: linking Ca2+ signalling to membrane dynamics. Nat Rev Mol Cell Biol.

[33] Liemann S, Huber R (1997). Three-dimensional structure of annexins. Cell Mol Life Sci.

[34] Hakobyan D, Gerke V, Heuer A (2017). Modeling of annexin A2-membrane interactions by molecular dynamics simulations. PLoS ONE.

[35] Miyagi A, Chipot C, Rangl M, Scheuring S (2016). High-speed atomic force microscopy shows that annexin V stabilizes membranes on the second timescale. Nat Nanotechnol.

[36] Hedhli N, Falcone DJ, Huang B, Cesarman Maus G, Kraemer R, Zhai H (2012). The annexin A2/S100A10 system in health and disease: emerging paradigms. J Biomed Biotechnol.

[37] Golczak M, Kirilenko A, Bandorowicz Pikula J, Pikula S (2001). N-and C-terminal halves of human annexin VI differ in ability to form low pH-induced ion channels. Biochem Biophys Res Commun.

[38] Kourie JI, Wood HB (2000). Biophysical and molecular properties of annexin-formed channels. Prog Biophys Mol Biol.

[39] Raynal P, Pollard HB (1994). Annexins: the problem of assessing the biological role for a gene family of multifunctional calcium- and phospholipid-binding proteins. Biochim Biophys Acta.

[40] Sheets EE, Giugni TD, Coates GG, Schlaepfer DD, Haigler HT (1987). Epidermal growth factor dependent phosphorylation of a 35-kilodalton protein in placental membranes. Biochemistry.

[41] He L, Ren M, Chen X, Wang X, Li S, Lin J (2014). Biochemical and immunological characterization of annexin B30 from *Clonorchis sinensis* excretory/secretory products. J Parasitol Res.

[42] Leow CY, Willis C, Osman A, Mason L, Simon A, Smith BJ (2014). Crystal structure and immunological properties of the first annexin from *Schistosoma mansoni*: insights into the structural integrity of the schistosomal tegument. FEBS J.

[43] Tararam CA, Farias LP, Wilson RA, de Cerqueira Leite LC (2010). *Schistosoma mansoni* Annexin 2: molecular characterization and immunolocalization. Exp Parasitol.

[44] de la Torre EE, Manzano Román R, Siles Lucas M, Pérez Sánchez R, Moyano JC, Barrera I (2012). Molecular and functional characterization of a *Schistosoma bovis* annexin: fibrinolytic and anticoagulant activity. Vet Parasitol.

[45] Popa SJ, Stewart SE, Moreau K (2018). Unconventional secretion of annexins and galectins. Semin Cell Dev Biol..

[46] Rescher U, Gerke V (2004). Annexins-unique membrane binding proteins with diverse functions. J Cell Sci.

[47] Dalli J, Consalvo AP, Ray V, Di Filippo C, D’Amico M, Mehta N (2013). Proresolving and tissue-protective actions of annexin A1-based cleavage-resistant peptides are mediated by formyl peptide receptor 2/lipoxin A_4_ receptor. J Immunol.

[48] Walther A, Riehemann K, Gerke V (2000). A novel ligand of the formyl peptide receptor: annexin I regulates neutrophil extravasation by interacting with the FPR. Mol Cell.

[49] McVoy LA, Kew RR (2005). CD44 and annexin A2 mediate the C5a chemotactic cofactor function of the vitamin D binding protein. J Immunol.

[50] Miskolci V, Klemm LC, Huttenlocher A (2021). Cell migration guided by cell-cell contacts in innate immunity. Trends Cell Biol.

[51] Drechsler M, de Jong R, Rossaint J, Viola JR, Leoni G, Wang JM (2015). Annexin A1 counteracts chemokine-induced arterial myeloid cell recruitment. Circ Res.

[52] Subedi L, Gaire BP, Kim S-Y, Parveen A (2021). Nitric oxide as a target for phytochemicals in anti-neuroinflammatory prevention therapy. Int J Mol Sci.

[53] Bogdan C (2015). Nitric oxide synthase in innate and adaptive immunity: an update. Trends Immunol.

[54] Gottstein B, Soboslay P, Ortona E, Wang J, Siracusano A, Vuitton D (2017). Immunology of alveolar and cystic echinococcosis (AE and CE). Adv Parasitol.

[55] Castro F, Cardoso AP, Gonçalves RM, Serre K, Oliveira MJ (2018). Interferon-gamma at the crossroads of tumor immune surveillance or evasion. Front Immunol.

[56] Siracusano A, Delunardo F, Teggi A, Ortona E (2012). Cystic echinococcosis: aspects of immune response, immunopathogenesis and immune evasion from the human host. Endocr Metab Immune Disord Drug Targets.

[57] Yoshimura A, Muto G. TGF-β function in immune suppression. In: Ahmed, R., Honjo, T. (eds) Negative co-receptors and ligands. Current Topics in Microbiology and Immunology, vol 350. Springer, Berlin, Heidelberg. 2011:127–147. 10.1007/82_2010_87 10.1007/82_2010_87 20680806

[58] Amri M, Aissa SA, Belguendouz H, Mezioug D, Touil-Boukoffa C (2007). In vitro antihydatic action of IFN-γ is dependent on the nitric oxide pathway. Interferon Cytokine Res.

