# Supplementary material for: Molecular characterization and functional implications on mouse peripheral blood mononuclear cells of annexin proteins from Echinococcus granulosus sensu lato
Source: Parasit Vectors. 2023 Oct 6;16:350. doi: 10.1186/s13071-023-05967-y (PMC10559496; doi:10.1186/s13071-023-05967-y)
Supplement: Supplementary file 1 — Additional file 1: Table S1. Primers for the PCR amplification of four EgANXBs. Table S2. Primers for the qRT-PCR amplification of four EgANXBs and GAPDH. Table S3. Primers for the qRT-PCR of cytokines. [file 13071_2023_5967_MOESM1_ESM.docx]

| **Supplementary Table 1** Primers for the PCR amplification of four *Eg*ANXBs | | | |
| --- | --- | --- | --- |
| Gene | Primers (5′-3′) | Restriction  Endonuclease | Reference Sequences WormBase ParaSite ID |
| *Eg*ANXB2 | F:CGAGCTCATGGCCAAAAACGCTC | *Sac*I | EgrG_000193700 |
|  | R:CCCTCGAGTCAAGATGCATTCAATAG | *Xho*I |  |
| *Eg*ANXB18 | F: CGGGATCCATGAGCATCAAAGTTACC | *Bam*HI | EgrG_000041200 |
|  | R: CGGAATTCTTACTGAGATCCGAGGAGAC | *Eco*RI |  |
| *Eg*ANXB20 | F: CGGATCCATGGCCACCGTGCATGCAGC | *Bam*HI | EgrG_000244000 |
|  | R: CCGGAATTCTTATTTATCCTCATGAACG | *Eco*RI |  |
| *Eg*ANXB23 | F: CGGATCCATGAATGGGCGTCCAACTATC | *Bam*HI | EgrG_000237700 |
|  | R: CCGGAATTCTTATGCATTCCCCTCTAC | *Eco*RI |  |
| The underlined parts indicate the restriction enzyme sites. | | | |

**Supplementary Table 2** Primers for the qRT-PCR amplification of four *Eg*ANXBs and *GAPDH*

| Gene | Primers (5′-3′) |
| --- | --- |
| *Eg*ANXB2 | F: TCCTACATAAGCGTTCTGCGTGAC |
|  | R: TTGGAGTTGCTCTGGCGTTGG |
| *Eg*ANXB18 | F: AGCACTTCAACGACGGTCTAAGC |
|  | R: TGAGATCCGAGGAGACGAACGAG |
| *Eg*ANXB20 | F: AACGACCTCTTCACCACAGTTGAG |
|  | R: TCGGTATTGCAGTGCGCCATG |
| *Eg*ANXB23 | F: GGTGGCGGAGATGAAGGCATG |
|  | R: CAACGGCGATGACCTGGATGAG |
| *GAPDH* | F: ACTCCGTCAATGTTGTCGCTGTC |
|  | R: GTCAATAACCAACTTGCCGCCATC |

**Supplementary Table 3** Primers for the qRT-PCR of cytokines

| Gene | Primers (5′-3′) |
| --- | --- |
| IL-10 | F: CTTGCACTACCAAAGCCACAAAGC |
|  | R: GTCAGTAAGAGCAGGCAGCATAGC |
| IFN-γ | F: CTGGAGGAACTGGCAAAAGGATGG |
|  | R: GACGCTTATGTTGTTGCTGATGGC |
| IL-17A | F: TGATGCTGTTGCTGCTGCTGAG |
|  | R: CACATTCTGGAGGAAGTCCTTGGC |
| TGF-β1 | F: ACCGCAACAACGCCATCTATGAG |
|  | R: GGCACTGCTTCCCGAATGTCTG |
| *GAPDH* | F: TGTGTCCGTCGTGGATCTGA |
|  | R: TTGCTGTTGAAGTCGCAGGAG |
